# The basal release of endothelium-derived catecholamines regulates the contractions of *Chelonoidis carbonaria* aorta caused by electrical-field stimulation

**DOI:** 10.1242/bio.057042

**Published:** 2021-01-20

**Authors:** José Britto-Júnior, Felipe Fernandes Jacintho, Rafael Campos, David Halen Araújo Pinheiro, Guilherme M. Figueiredo Murari, Valéria B. de Souza, André A. Schenka, Fabíola Z. Mónica, Ronilson Agnaldo Moreno, Edson Antunes, Gilberto De Nucci

**Affiliations:** 1 Faculty of Medical Sciences, Department of Pharmacology, University of Campinas (UNICAMP), Campinas 13083-894, Brazil; 2 Department of Physiology, Superior Institute of Biomedical Sciences, Ceará State University (UECE), Fortaleza 60714-903, Brazil; 3 Department of Pharmacology, Institute of Biomedical Sciences, University of São Paulo, São Paulo 05508-060, Brazil

**Keywords:** LC-MS-MS, Tortoise, Vessel, ODQ, L-NAME, Tyrosine hydroxylase

## Abstract

The contractions of *Chelonoidis carbonaria* aortic rings induced by electrical field stimulation (EFS) are not inhibited by blockade of the voltage-gated sodium channels by tetrodotoxin but almost abolished by the α1/α2-adrenoceptor antagonist phentolamine. The objective of this study was to identify the mediator(s) responsible for the EFS-induced contractions of *Chelonoidis carbonaria* aortic rings. Each ring was suspended between two wire hooks and mounted in isolated 10 ml organ chambers filled with oxygenated and heated Krebs-Henseleit's solution. Dopamine, noradrenaline and adrenaline concentrations were analysed by liquid chromatography coupled to tandem mass spectrometry. The contractions caused by dopamine and EFS were done in absence and presence of the nitric oxide (NO) synthesis inhibitor L-NAME, the NO-sensitive guanylyl cyclase inhibitor ODQ, the D1-like receptor antagonist SCH-23390, the D2-like receptor antagonists risperidone, quetiapine, haloperidol, and the tyrosine hydroxylase inhibitors salsolinol and 3-iodo-L-tyrosine. Basal concentrations of dopamine, noradrenaline and adrenaline were detected in Krebs-Henseleit solution containing the aortic rings. The catecholamine concentrations were significantly reduced in endothelium-denuded aortic rings. L-NAME and ODQ significantly potentiated the dopamine-induced contractions. The D2-like receptor antagonists inhibited the EFS-induced contractions of the aortic rings treated with L-NAME, whereas SCH 23390 had no effect. Similar results were observed in the contractions induced by dopamine in L-NAME treated aortic rings. These results indicate that catecholamines released by endothelium regulate the EFS-induced contractions. This may constitute a suitable mechanism by which reptilia modulate specific organ blood flow distribution.

This paper has an associated First Person interview with the first author of the article.

## Abstract (precis)

**Summary:** *Chelonoidis carbonaria* aortic rings release endothelium-derived catecholamines that modulate vascular smooth muscle reactivity.

## INTRODUCTION

It is well established that endothelial cells modulate vascular reactivity through the release of mediators such as prostacyclin (Moncada et al., 1976), nitric oxide (Furchgott and Zawadzki, 1980) and endothelin (Yanagisawa et al., 1988). Catecholamines modulate vascular tonus through the actions on α- and β-adrenoceptors (Ahlquist, 1948); however, the production and release of catecholamines are associated with the existence of nerve terminals on vessels (Kadowitz et al., 1976; Matsuyama et al., 1985).

Electrical-field stimulation (EFS) is a technique in which an electrical stimulus is applied uniformly to an isolated tissue in short pulse widthwaves (Paterson, 1965; Bevan, 1962). EFS is commonly used in protocols evaluating adrenergic (Campos et al., 2019a,b; Dail et al., 1987), cholinergic (De Oliveira et al., 2019) and non-adrenergic non-cholinergic events (Ignarro et al., 1990; De Oliveira et al., 2003). Tetrodotoxin is considered an inhibitor of nerve terminal stimulation, since it blocks voltage-sensitive sodium channels (Narahashi et al., 1964).

Electrical-field stimulation causes aortic contractions of the tortoise *Chelonoidis carbonaria*, but these responses are not inhibited by tetrodotoxin, indicating they are not due to nerve terminal stimulation (Campos et al., 2020). Interestingly, these EFS-induced aortic contractions are reduced by either the α-adrenoceptor antagonist phentolamine or by endothelium removal (Campos et al., 2020), suggesting a potential modulatory role for endothelium-derived catecholamines. Similar observations have been reported for EFS-induced aortic contractions of the snakes *Crotalus durissus terrificus*, *Bothrops jararaca* (Campos et al., 2018a) and *Panterophis guttatus* (Campos et al., 2018b), as well as of the human umbilical cord vessels (Britto-Júnior et al., 2020a). Since immunohistochemistry failed to identify nerve terminals in *Chelonoidis carbonaria* aortae (Campos et al., 2020), the results indicate a non-neuronal source of catecholamine synthesis. Interestingly, the enzyme tyrosine hydroxylase, responsible for catalyzing the conversion of L-tyrosine to L-DOPA, was identified only in the endothelial cells from *Chelonoidis carbonaria* aorta (Campos et al., 2020) and from both human umbilical artery and human umbilical vein (Britto-Junior et al., 2020b). The inhibition by phentolamine of EFS-induced contractions in both tortoise (Campos et al., 2020) and umbilical cord vessels (Britto-Júnior et al., 2020a) was observed only at high concentrations of this adrenoceptor antagonist, suggesting that it may be acting on a different population of receptors. In addition, a basal endothelium-derived dopamine release was identified by tandem mass spectrometry in human umbilical cord vessels and use of the dopamine D2-like receptor antagonist haloperidol reduced the EFS-induced contraction in human umbilical cord artery and vein (Britto-Junior et al., 2020b).

In this manuscript, the nature of the mediators released by endothelial cells of aortic rings of *Chelonoidis carbonaria* was identified by liquid chromatography coupled to tandem mass spectrometry (LC-MS-MS), followed by a pharmacological characterization of the EFS-induced contractions in *Chelonoidis carbonaria* aortic rings *in vitro*.

## RESULTS

### Determination of catecholamine concentrations by LC-MS-MS

**Fig. 1.**
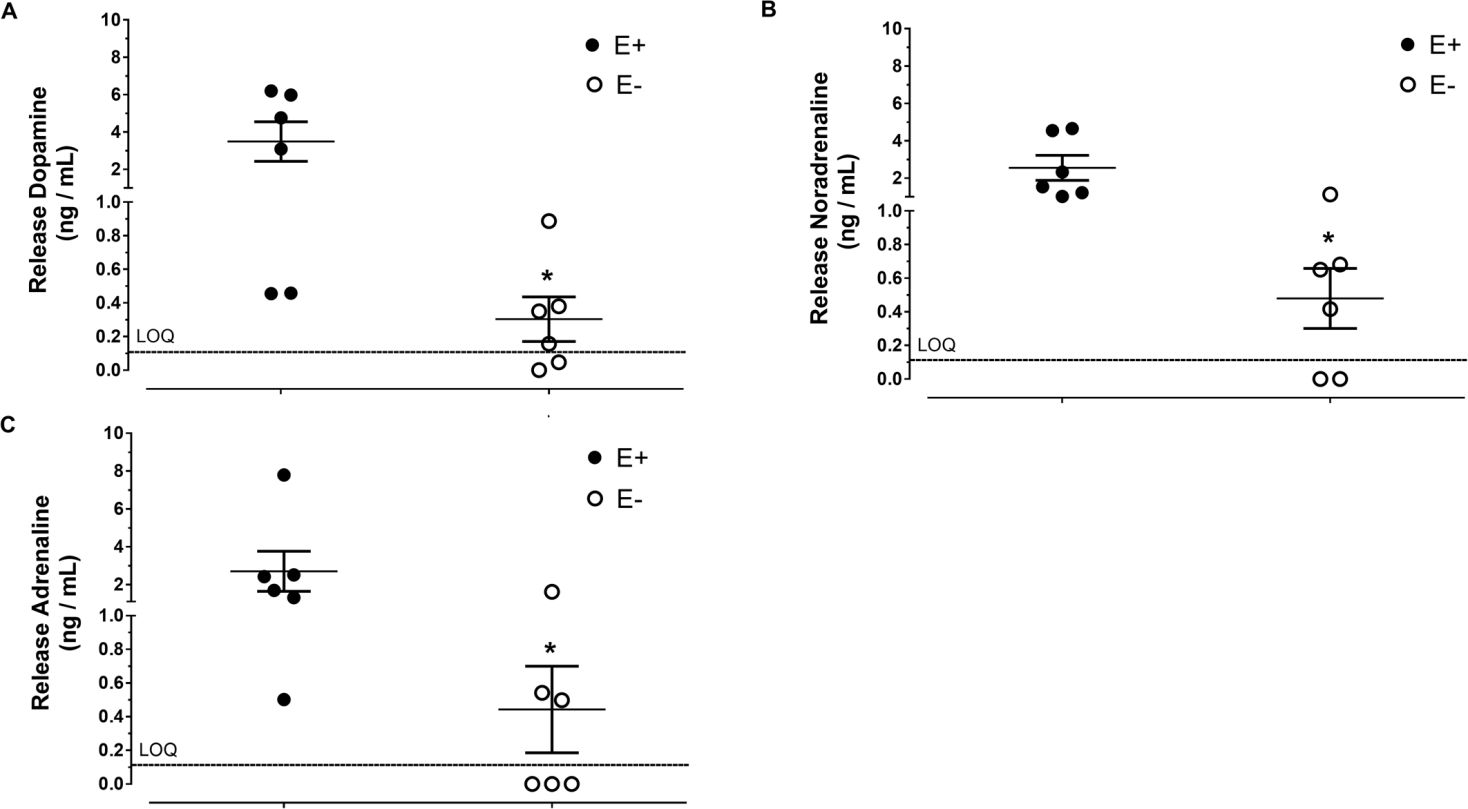
**Basal release of catecholamines in endothelium-intact aortic rings of *Chelonoidis carbonaria*.** The basal release of dopamine (A), noradrenaline (B) and adrenaline (C) in Krebs-Henseleit solution after 30 min incubation with endothelium-intact (E+; *n*=6/6) and endothelium-denuded aortic rings (E- *n*=6/6). *P*<0.05 compared with E- preparations.

Dopamine, noradrenaline and adrenaline calibration curves were linear for concentrations of 0.1-10.0 ng/ml, with a correlation coefficient greater than 0.99. The lower limit of quantification was 0.1 ng/ml. Dopamine, noradrenaline and adrenaline concentrations were above the limit of quantification in the Krebs-Henseleit solution of all six of the aortic rings with endothelium intact. The basal releases of catecholamines were significantly reduced in endothelium-denuded aortic rings (*n*=6/6; Fig. 1).

### Effect of L-NAME and ODQ in aortic rings

**Fig. 2.**
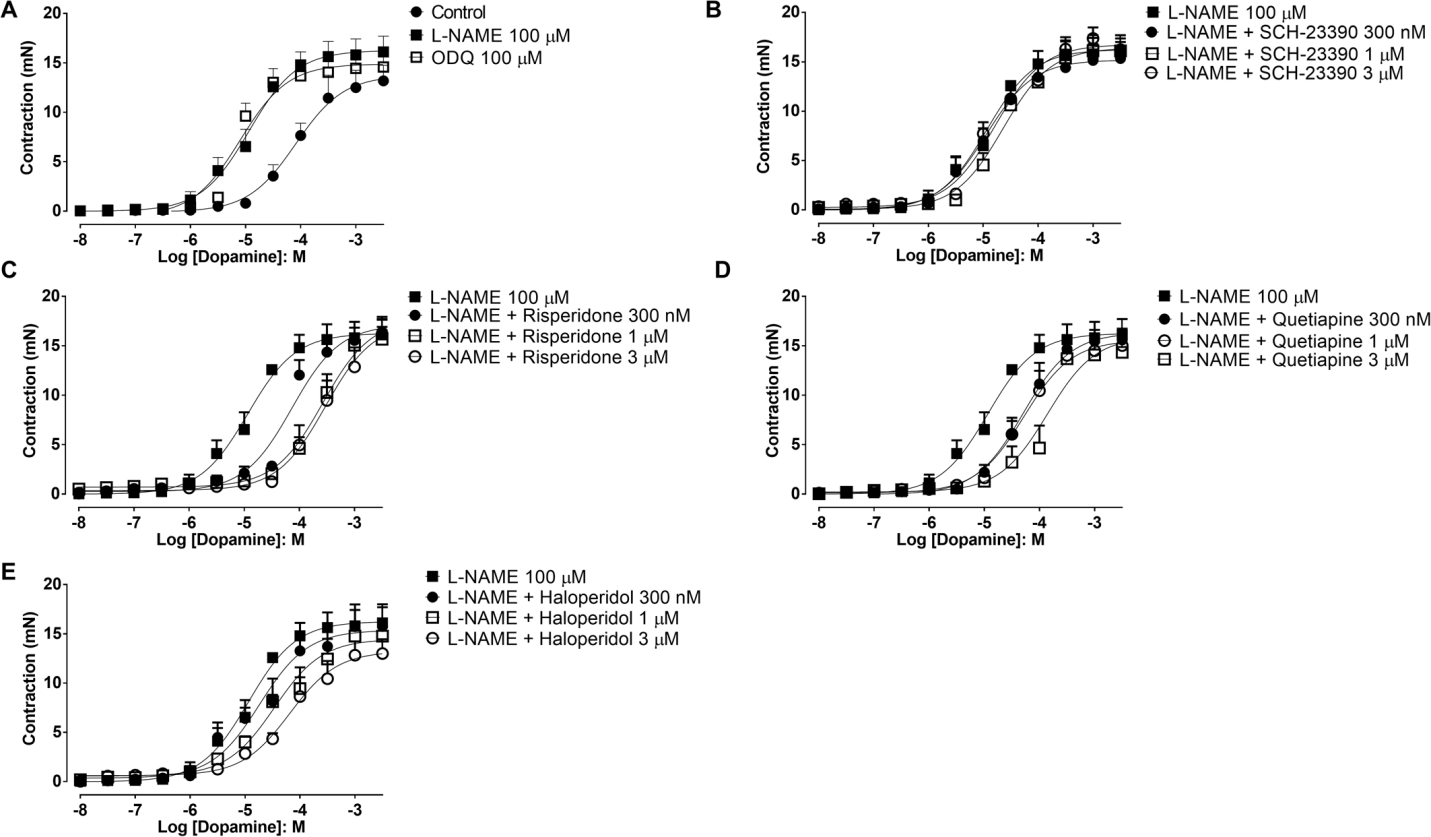
**Concentration-response curves to dopamine in *Chelonoidis carbonaria* aortic rings.** Cumulative concentration-response curves to dopamine in *Chelonoidis carbonaria* aortic rings was performed in presence and absence of L-NAME (100 µM; *n*=5/6) and ODQ (100 µM; *n*=5/5; panel (A). Concentration-response curves to dopamine in L-NAME-pretreated preparations (100 µM; *n*=5/6) were also performed in presence and absence of the D1-like receptors antagonist SCH 23390 (0.3, 1 and 3 µM; *n*=4/5; B) and the D2-like receptors antagonists risperidone (0.3, 1 and 3 µM; *n*=4/5; C), quetiapine (0.3, 1 and 3 µM; *n*=4/5; D) and haloperidol (0.3, 1 and 3 µM; *n*=4/5; E). Data are expressed as mean±s.e.m.

Dopamine caused concentration-dependent contractions of endothelium-intact aortic rings (E_max_ 13.2±1.6 mN; pEC_50_ 4.0±0.1, *n*=4/5; Fig. 2A). Incubation with L-NAME (100 µM) caused a significant leftward shift of the concentration-response curves to dopamine (pEC_50_ 5.1±0.2, *P*<0.05) accompanied by an increase in E_max_ value (16.1±1.6 mN, *n*=5/6; Fig. 2A, *P*<0.05). Likewise, incubation of the preparations with ODQ (100 µM) caused a significant (*P*<0.05) leftward shift (pEC_50_ 5.1±0.1) and a significant increase of the E_max_ value (14.6±1.4 mN, *n*=5/5; Fig. 2A).

### Evaluation of dopamine receptors in aortic rings

In L-NAME (100 µM)-treated aortic rings, the dopamine D1-like receptor antagonist SCH-23390 caused no significant shifts in the dopamine-induced aortic contractions (pEC_50_ 4.9±0.2, 4.6±0.1 and 4.7±0.1 for 0.3, 1 and 3 μM, respectively, *n*=4/5) compared with L-NAME alone (pEC50 5.1±0.2; Fig. 2B).

The dopamine D2-like receptor antagonist risperidone caused significant concentration-dependent rightward shifts of the concentration-dependent dopamine contractions in L-NAME-treated aortic rings (pEC_50_ 4.1±0.1, 3.6±0.1 and 3.1±0.3 for 0.3, 1 and 3 µM, respectively; *n*=4/5) compared with L-NAME alone (pEC_50_ 5.1±0.2, *n*=5/6; Fig. 2C). The E_max_ values were not significantly changed by risperidone (Fig. 2C).

**Fig. 3.**
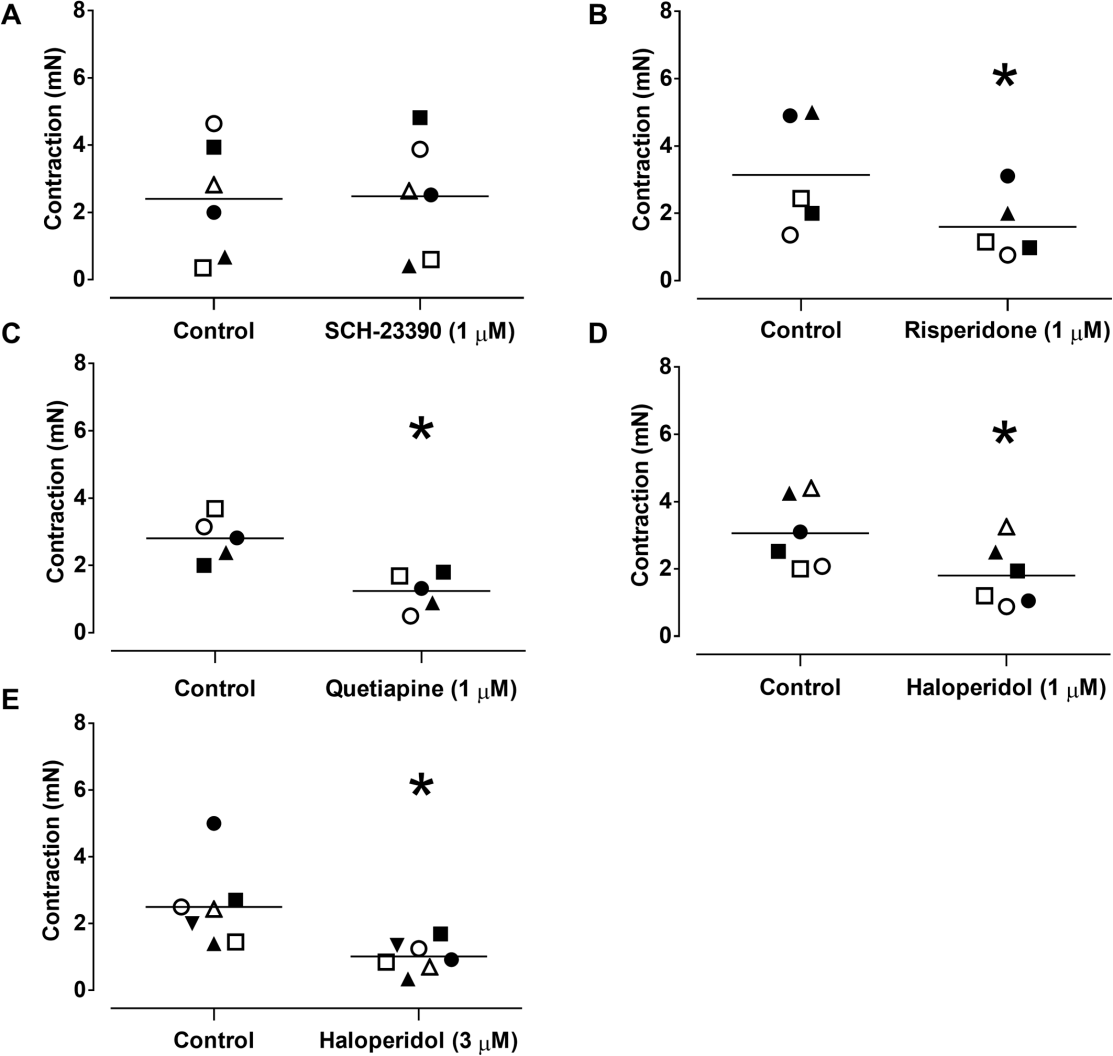
**Effects of D1-like and D2-like receptor antagonists on EFS-induced contractions of aortic rings of *Chelonoidis carbonaria*.** Scatter plots show the individual values of the effects of the D1-like receptor antagonist SCH-23390 (*n*=4/6; 1 µM; A) and the D2-like receptor antagonists risperidone (1 µM; *n**=***4/5; B), quetiapine (1 µM; *n*=4/5; C) and haloperidol (1 µM; *n*=4/6; D and 3 µM; *n =* 5/7; E) on EFS (16 Hz)-induced contractions of aortic rings pretreated with L-NAME (100 µM). *P<0.05. Each individual symbol represents a ring before and after treatment.

**Fig. 4.**
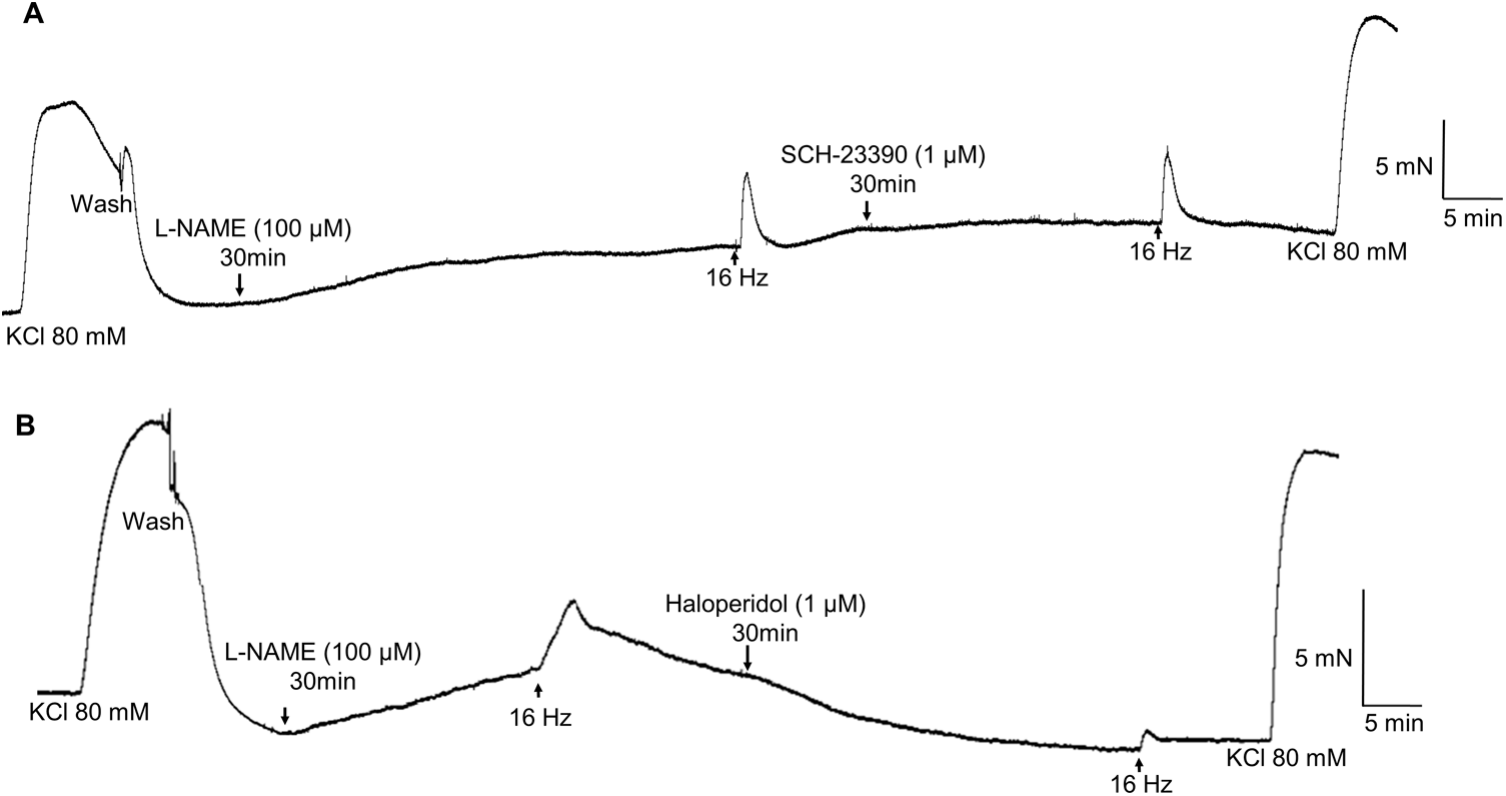
**Representative tracing of the effect of the D1-like receptor antagonist SCH 23390 (1 µM; *n*= 4/6) and the D2-like receptor antagonist haloperidol (1 µM; *n***=**4/6) on EFS (16 Hz)-induced contractions of aortic rings of *Chelonoidis carbonaria* pretreated with L-NAME (100 µM).**

The dopamine D2-like receptor antagonists quetiapine (0.3, 1 and 3 µM) and haloperidol (1 and 3 µM) also caused significant rightward shifts of the concentration-dependent dopamine contractions in L-NAME-treated aortic rings without affecting the E_max_ values (Table 1; Fig. 2D,E, respectively). In L-NAME-treated aortic rings, the dopamine D1-like receptor antagonist SCH-23390 (1 µM, *n*=4/6) had no significant effect on the EFS-induced contractions of aortic rings (2.4±0.7 and 2.5±0.7 mN at 16 Hz, for control and SCH-23390, respectively; Figs 3A and 4A).

**Table 1.**
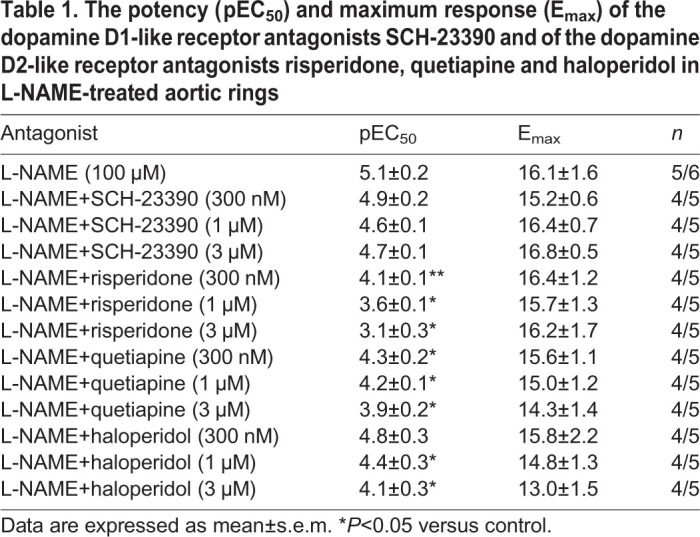
**The potency (pEC_50_) and maximum response (E_max_) of the dopamine D1-like receptor antagonists SCH-23390 and of the dopamine D2-like receptor antagonists risperidone, quetiapine and haloperidol in L-NAME-treated aortic rings**

The dopamine D2-like receptor antagonist risperidone (1 µM, *n*=4/6) significantly (*P*<0.05) reduced the EFS (16 Hz)-induced contractions in L-NAME-treated aortic rings (3.1±0.8 and 1.6±0.4 mN for control and risperidone, respectively; Fig. 3B). Quetiapine (1 µM, *n*=4/5) also significantly reduced (*P*<0.05) the EFS-induced contractions (2.8±0.3 and 1.2±0.2 mN for control and quetiapine, respectively; Fig. 3C). Similar reductions were observed with haloperidol at both 1 µM (3.1±0.4 and 1.8±0.3 mN for control and test, respectively; Figs 3D and 4B) and 3 µM (2.5±0.5 and 1.0±0.2 for control and test, respectively, *n*=5/7; Fig. 3E).

### Effect of L-NAME and risperidone on basal tonus of aortic rings

**Fig. 5.**
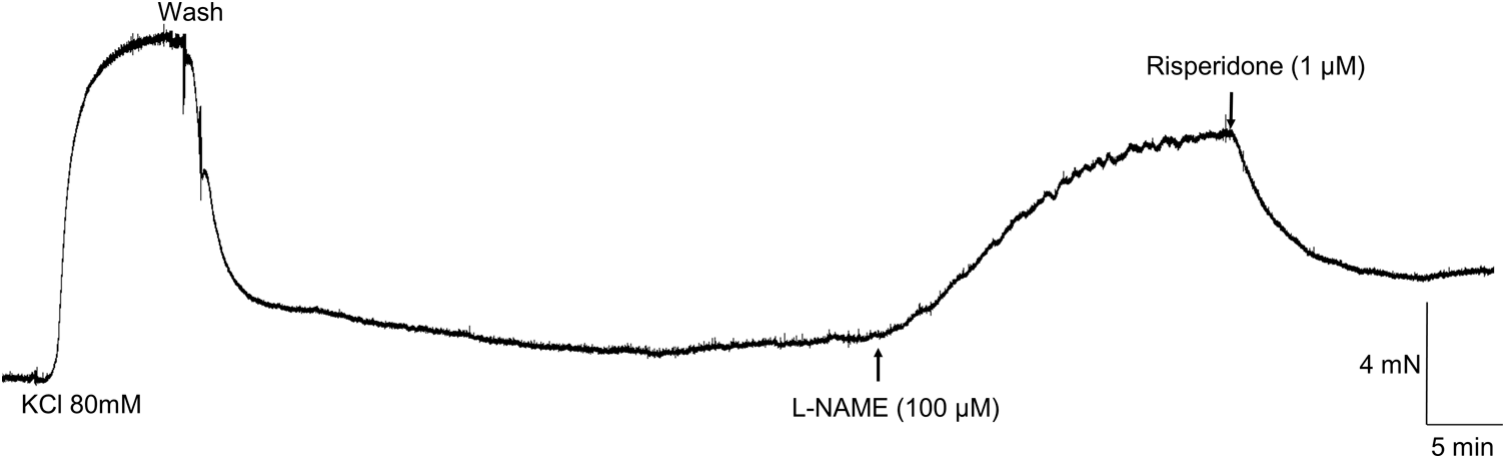
**Representative tracing showing the reversal by risperidone (1 µM; *n*=5/5 of the elevated tonus induced by L-NAME (100 µM) in aortic rings of *Chelonoidis carbonaria.***

In L-NAME (100 µM)-treated aortic rings, the basal tonus was increased in 10/18 out of 10/32 aortic rings. The elevated basal tonus induced by L-NAME (5.5±1.6 mN) was promptly reversed by risperidone (1 µM; reduction to 3.6±1.0 mN) in all aortic rings tested (*n*=5/5; Fig. 5).

### Effect of tyrosine hydroxylase inhibition with salsolinol and 3-Iodo-L-tyrosine

**Fig. 6.**
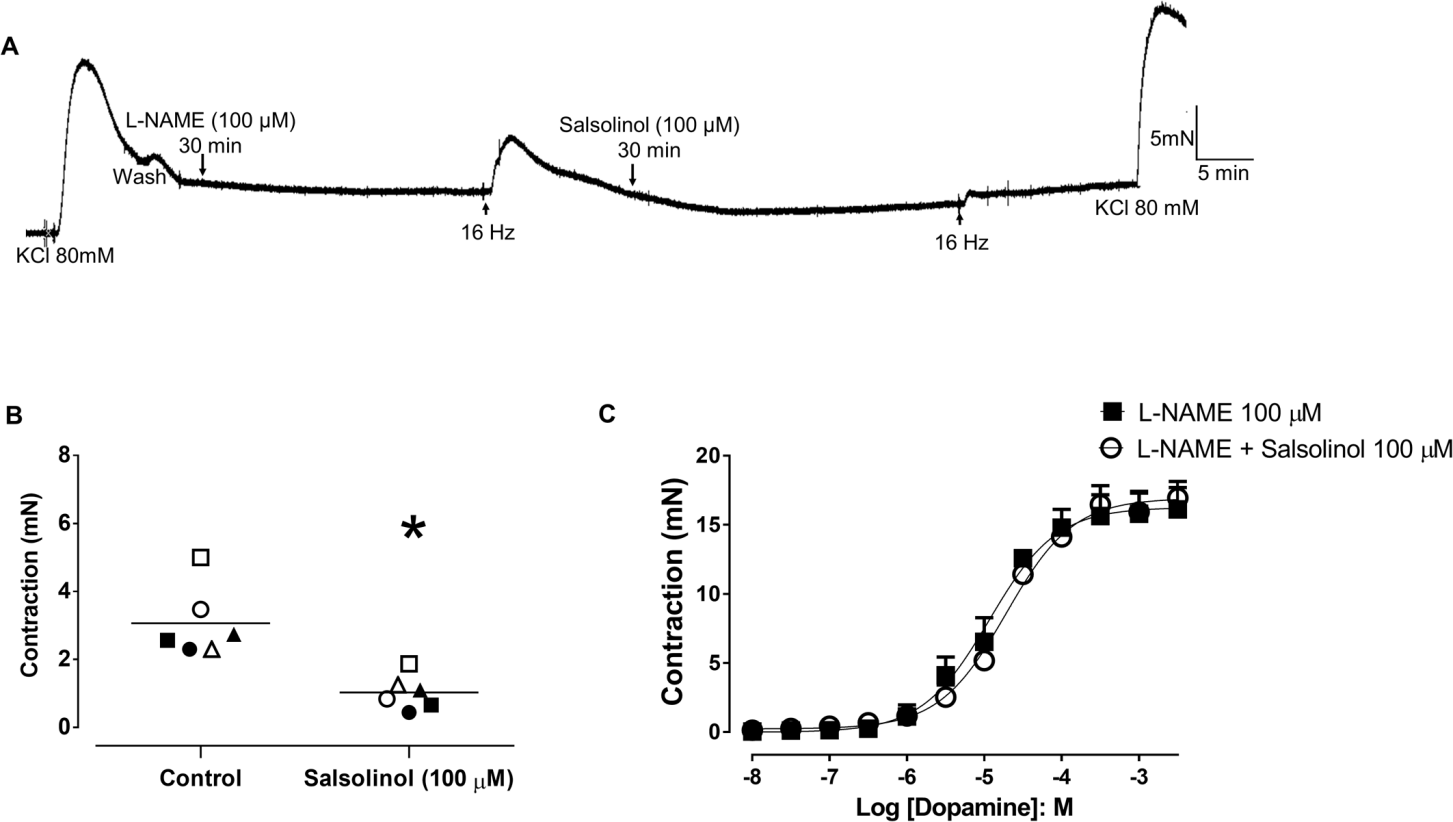
**Effect of the tyrosine hydroxylase inhibitor salsolinol on EFS-induced contraction of aortic rings of *Chelonoidis carbonaria*.** (A) Representative tracing displaying the inhibitory effect of salsolinol (100 µM) on EFS (16 Hz)-induced contraction of aortic rings pretreated with L-NAME (100 µM; *n*=4/6). (B) Scatter plots of individual values and mean values ±s.e.m. of the EFS-induced contractions of L-NAME (100 µM)-treated preparations in the presence and the absence of salsolinol (100 µM; *n*=4/6). (C) Cumulative concentration-response curves to dopamine in aortic rings pretreated with L-NAME (100 µM; *n*=5/6) in the presence and absence of the salsolinol (100 µM; *n*=5/5). *P<0.05 compared with control. Each individual symbol represents a ring before and after treatment.

The EFS (16 Hz)-induced aortic contractions in L-NAME (100 µM)-treated preparations were significantly reduced (*P*<0.05) by incubation with the tyrosine hydroxylase inhibitor salsolinol (100 μM; 3.1±0.4 and 1.0±0.2 mN for control and salsolinol, respectively; Fig. 6A,B; *n*=4/6). On the other hand, salsolinol (100 µM, *n*=4/5) had no effect on the dopamine-induced contractions of L-NAME-treated aortic rings (E_max_ 16.1±1.6 and 16.9±1.2 mN and pEC_50_ 5.1±0.2 and 4.7±0.1 for L-NAME alone and salsolinol, respectively; Fig. 6C).

**Fig. 7.**
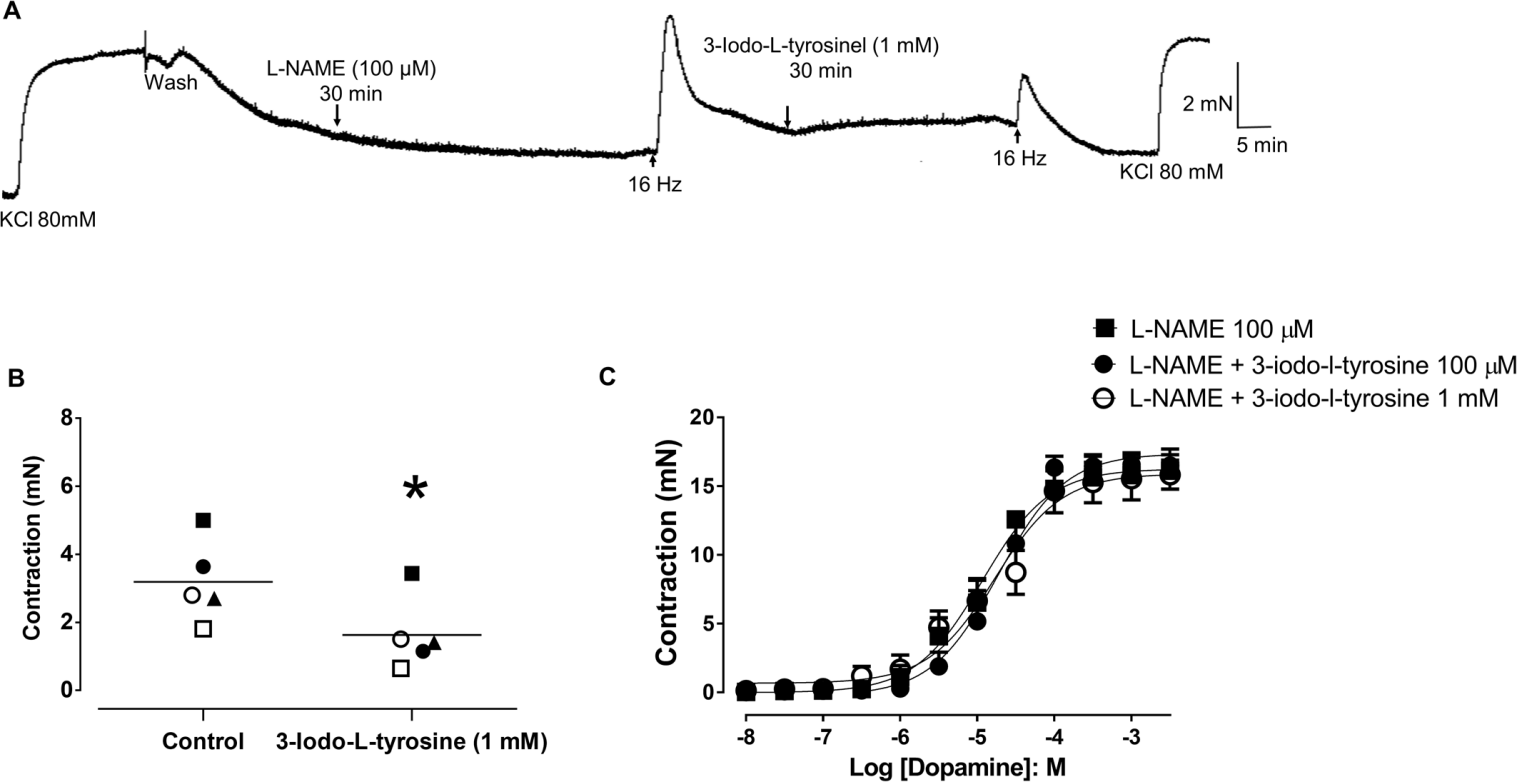
**Effect of the tyrosine hydroxylase inhibitor 3-iodo-tyrosine on EFS-induced contraction of aortic rings of *Chelonoidis carbonaria*.** (A) Representative tracing of the inhibitory effect of 3-iodo-L-tyrosine (1 mM) on EFS (16 Hz)-induced contraction of aortic rings pretreated with L-NAME (100 µM; *n*=3/5). (B) shows scatter plots of individual values and mean values ±s.e.m. of the EFS-induced contraction in aortic rings pretreated with L-NAME (100 µM) in the presence and the absence of 3-iodo-L-tyrosine (1 mM; *n*=3/5). (C) Cumulative concentration-response curve to dopamine in aortic rings pretreated with L-NAME (100 µM; *n*=5/6) in the presence and absence of the 3-iodo-L-tyrosine (0.1 and 1 mM; *n*=5/5 for each curve). *P <0.05 compared with control. Each individual symbol represents a ring before and after treatment.

The EFS (16 Hz)-induced contractions of L-NAME-treated aortic rings were also significantly reduced (*P*<0.05) by incubation with the tyrosine hydroxylase inhibitor 3-Iodo-L-tyrosine (1 mM; Fig. 7A,B; *n*=3/5). The 3-iodo-L-tyrosine had no effect on the dopamine-induced contractions of L-NAME-treated aortic rings (E_max_ 16.5±0.8 and 15.8±1.0 mN; pEC_50_ 4.6±0.1 and 4.9±0.1 for 0.1 and 1 mM of iodo-L-tyrosine, respectively; *n*=5/5) compared with L-NAME alone (E_max_ 16.1±1.6 mN, pEC_50_ 5.1±0.2, *n*=5/6; Fig. 7C).

### Immunohistochemistry

**Fig. 8.**
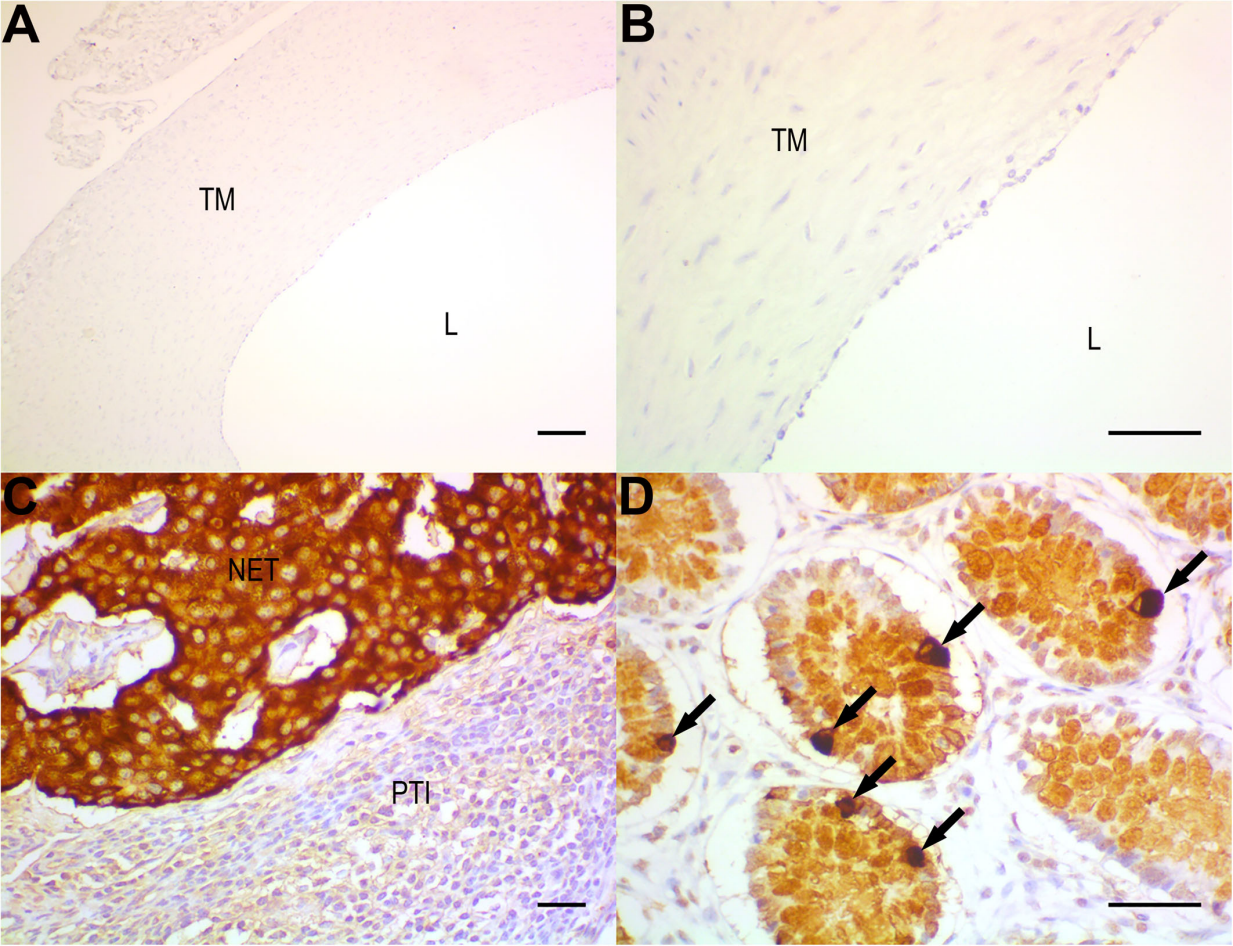
**Chromogranin A detection by immunohistochemistry.** (A) lack of positivity for chromogranin A (CgA) in Chelonoidis aortic smooth muscle cells of the tunica media (TM) and in endothelial cells lining the lumen (L), low-power field (100X, original magnification); (B) same as in previous photomicrograph, at high-power field (400X). (C) Strong and diffuse positivity for CgA in a neuroendocrine tumor (NET) of the appendix, serving as a positive control. (D) Strong positivity also seen in scattered chromaffin cells (arrows), in a normal intestinal mucosae specimen (another positive control tissue). Immunoperoxidase, scale bars: 100 μm in (A) and (C); 50 μm in (B) and (D). PTI, peritumoral inflammation lacking positivity for chromogranin A.

Fig. 8A and B show that there was an absence of Chromogranin A staining (a biomarker for chromaffin cells) in all sections of Chelonoidis aortae that were tested. Positive controls demonstrated the presence of Chromogranin A staining in neuroendocrine tumor and normal chromaffin cells from the colon (Fig. 8C,D).

## DISCUSSION

The results presented here clearly demonstrate, for the first time in the tortoise, that *Chelonoidis carbonaria* aortae have a basal release of dopamine, noradrenaline and adrenaline, as identified by tandem mass spectrometry, and the amount released is significantly reduced by endothelium-removal. Basal release of endothelium-derived catecholamines also occur in human umbilical vessels (Britto-Júnior et al., 2020b).

The contractions induced by EFS in the aortic rings were only inhibited by the non-selective α-adrenergic blocker phentolamine at high concentrations. The finding that the α1 antagonist prazosin (Agrawal et al., 1984) and the α2 antagonist idazoxan (Doxey et al., 1984) had no effect on the contractions of *Chelonoidis carbonaria* aortic rings induced by EFS indicated that the inhibition by phentolamine is unlikely to be due to its action on α-adrenoceptors (Campos et al., 2020). Phentolamine also acts as an antagonist of dopaminergic receptors, since it displaces ^3^H-haloperidol binding at concentrations above 2 µM in calf brain membranes (Burt et al., 1976). In our study, the contractions induced by EFS were inhibited by the D_2_-like receptor antagonists risperidone, quetiapine and haloperidol, but not affected by the D1-like receptor antagonist SCH-23390 (Billard et al., 1984). Dopaminergic receptors in vascular beds have been identified *in vitro* by radioligand-receptor binding and autoradiographic techniques. The localization of dopamine-1 (D_1_) (Amenta and Ricci, 1990) and dopamine-2 (D_2_) receptors have been assessed in smooth muscle tissue of rat cerebral, mesenteric and renal arteries (Amenta et al., 1990). The contraction of *Chelonoidis carbonaria* aortic rings induced by dopamine was blocked by D_2_-like antagonists, indicating the presence of D_2_-like receptors. Furthermore, the EFS-induced contractions were also blocked by D2-like receptor antagonists, indicating that release of dopamine plays a major role on this phenomenon. The contractions induced by EFS in human umbilical artery and vein are also blocked by D_2_-like receptor antagonists, but not affected by the D_1_-receptor antagonist SCH-23390 (Britto-Junior et al., 2020b). The inhibition of EFS-induced contractions by the D_2_-like receptor antagonist haloperidol reveals an important modulatory role of the endothelium-derived dopamine, acting as a vasoconstrictor through the D2-like receptors. It is interesting that both domperidone and haloperidol applied as ophthalmic solutions in a rabbit ocular hypertensive model produced a marked increase of ocular blood flow (Chiou and Chen, 1992). It is important to mention that although endothelial cells are not considered excitable cells, they do express voltage-gated potassium channels (Félétou, 2011). Adams and Hill (2004) report that in endothelial cells (including in human capillaries), a fast-activating transient outward potassium current has been observed similar to that of vascular smooth muscle cells showing the characteristics of A-type potassium currents. Our results indicate that endothelial cells present a basal release of catecholamines but whether EFS induces further release of these mediators, remains to be further investigated and the data presented here only provides evidence that endothelial catecholamines modulate EFS-induced contractions. Although the heart output is defined as the product of heart rate and stroke volume, the pumping function of the heart has been considered to have a minor role in the determination of cardiac output (Guyton, 1981). The systemic outflow is primarily controlled by a balance of arterial vasodilatation (regulation of systemic conductance) and venous constriction (regulation of vascular capacitance; Joyce and Wang, 2020). Indeed, the heart output was largely unaffected by increase in the heart rate of electrically paced subjects (Ross et al., 1965). Patients who where subjected to heart transplantation present extrinsic heart denervation caused by axonal Wallerian degeneration due to surgical interruption of the parasympathetic vagal neurons and the intrinsic post-ganglionic sympathetic nerve fibers traveling from the stellate ganglia to the myocardium (Awad et al., 2016). Afferent and efferent denervation of the transplanted organs in heart-lung transplanted patients is caused by the interruption of the central connections from the low-pressure receptors in the atria and pulmonary veins (Jamieson et al., 1984). In a study comparing eight healthy heart-lung transplant recipients with eight normal subjects matched for age and sex revealed that the transplant group had significantly higher heart frequency and diastolic blood pressure (Banner et al., 1990). Interestingly, the increase of both heart frequency and diastolic pressure during head-up tilt were similar in the two groups. Baseline levels of noradrenaline and adrenaline were also similar in the two groups; however, during head-up tilt, plasma noradrenaline levels increased to a significantly greater extent in the transplant group as compared to the control group. It is clear from the above that the catecholamines are doing their job, and that they are not coming from the denervated adrenergic branches in the heart.

What is the possible physiological role(s) of endothelium-derived catecholamines in reptilia? Acute anoxic exposure of the turtle heart *Chrysemys scripta in situ* is accompanied by a weak negative chronotropic effect at both 5°C and 15°C (Farrell et al., 1994). An elevation of plasma catecholamine levels has been also associated to anoxia (Wasser and Jackson, 1991). The remarkable cardiovascular down-regulation that accompanies long periods of cold anoxia is characterized by a substantial increase in the systemic peripheral resistance, probably reflecting a prioritization of regional blood flow distribution (Hicks and Farrell, 2000). Indeed, α-adrenergic vasoactivity does contribute to blood flow regulation to the liver and shell during anoxic submergence at 5°C in the turtle *Trachemys scripta* (Stecyk et al., 2004). The differential release of catecholamines may be a suitable mechanism by which reptilia have specific organ blood flow distribution.

The basal release of dopamine, noradrenaline and adrenaline by *Chelonoidis carbonaria* aorta endothelial cells modulates EFS-induced contractions and endothelium-derived catecholamines acting on D2-like receptors may constitute a suitable mechanism for local control of blood flow in reptilia. It is known that large arteries, although capable of constricting and dilating, serve virtually no role in the regulation of pressure and blood flow under normal physiological conditions (Goodwill et al., 2017). However, what is being proposed is that endothelium-derived catecholamines will do that; endothelium-derived catecholamines should occur in all vessels, including the microcirculation. It is interesting that D2-receptors have been identified in rabbit pulmonary capillary microcirculation (Bruzzone et al., 2002).

Another possible source of extra-neuronal catecholamines is chromaffin cells. Chromaffin cells (neuroendocrine cells) grouped together make up paraganglia and are linked to both the visceral nervous system and the digestive tract. They can be distinguished into two categories: adrenal (i.e. the adrenal medulla) and extra-adrenal (Knottenbelt et al., 2015; La Perle and Dintzis, 2018). Interestingly, other non-mammal vertebrates have been shown to possess these cells associated with the autonomic system alongside the presence being in cardiac and vascular tissues, including the intercostal arteries and the azygous vein (Scheuermann, 1993; Nilsson, 2010). Nilsson, in particular, reported histological and histochemical evidence of chromaffin cells in lungfish heart and vascular walls (Nilsson, 2010). Until now, Chromogranin A and synaptophysin are considered reliable immunohistochemical markers for neuroendocrine/chromaffin differentiation (Kyriakopoulos et al., 2018). Despite positive controls undoubtedly showing the presence of chromogranin A, no chromogranin A staining was observed in any of the aortic ring tissues tested, indicating that these cells are not present in *Chelonoidis carbonaria* aortae, and thus cannot be responsible for the catecholamine release detected in this study.

## MATERIALS AND METHODS

### Animals

The experimental protocol using *Chelonoidis carbonaria* of either sex (weight varied from 2 to 7 kg) were authorized by the Institutional Animal Care and Use Committee (CEUA/UNICAMP: 3907-1, respectively) in compliance with the ARRIVE guidelines. The use of *Chelonoidis carbonaria* was approved by the Brazilian Institute for Environment (Sisbio; number 20910), and the tortoises were supplied by the Tietê Ecological Park (São Paulo, SP, Brazil).

### Chemical and reagents

Adrenaline, acetylcholine, noradrenaline, dopamine, adenosine 5′-triphosphate (ATP), N^ω^-Nitro-L-arginine methyl ester hydrochloride (L-NAME), H-[1,2,4]Oxadiazolo[4,3-a]quinoxalin-1-one (ODQ), 3-iodo-tyrosine, salsolinol and SCH-23390 were purchased from Sigma-Aldrich Chemicals Co. (St Louis, MO, USA). Risperidone, quetiapine and haloperidol were acquired from Nallin Farmácia e Manipulação Ltda (Itatiba-SP, Brazil). Dopamine-d3 hydrochloride, DL-noradrenaline-d6 hydrochloride and adrenaline-d6 hydrochloride were acquired from CDN Isotopes (Point Claire, Canada). Aluminium oxide was purchased from Dinamica Quimica Contemporanea Ltda (Indaiatuba-SP, Brazil). Sodium chloride (NaCl), potassium chloride (KCl), calcium chloride (CaCl_2_), magnesium sulfate (MgSO_4)_, sodium bicarbonate (NaHCO_3_), potassium phosphate monobasic (KH_2_PO_4_), and glucose were bought from Merck KGaA (Darmstadt, Germany). Acetonitrile was obtained from J.T Baker (Phillipsburg, NJ, USA) and formic acid (HPLC grade) was purchased from Mallinckrodt (St. Louis, MO, USA).

### Aortic ring preparations and isometric tension recordings

The tortoises were anesthetized with ketamine and propofol (40 mg/kg IM and 15 mg/kg IV, respectively) after sedation with midazolam (2 mg/kg; IM). The animals were euthanized by exsanguination. A segment of dorsal aorta was removed and immediately placed in oxygenated (95%O_2_/5%CO_2_) Krebs-Henseleit solution at 27°C. Subsequently, aortic rings (3 mm) were suspended vertically between two metal hooks in 10 ml organ baths containing Krebs-Henseleit solution (mM): NaCl (118), KCl (4.7), CaCl2 (2.5), MgSO4 (1.2), NaCO3 (25), KH2PO4 (1.2) and glucose (5.6), gassed with a mixture of 95% O2: 5% CO2 (pH 7.4) at 27°C, since it is the temperature often used for reptile tissue experiments (Stephens, 1984; Miller and Vanhoutte, 1986; Campos et al., 2019a,b). Isometric force was recorded using a PowerLab 400TM data acquisition system (Software Chart, version 7.0, AD Instrument, MA, USA). The tissues were allowed to equilibrate for 1 h before starting the experiments.

### Concentration-response curves to dopamine

Dopamine-induced concentration-dependent contractions were performed in endothelium-intact aortic rings in the absence and in the presence of the NO synthase inhibitor L-NAME (100 µM) and the NO-sensitive inhibitor of the guanylyl cyclase ODQ (100 µM). In L-NAME-treated aortic rings, dopamine-induced concentration-dependent contractions were also performed in the presence of the D1-like receptor antagonist SCH-23390 (0.3, 1 and 3 μM) and the D2-like receptor antagonists (risperidone, quetiapine and haloperidol; 0.3, 1 and 3 μM each), as well as of the tyrosine hydroxylase inhibitors salsolinol (100 μM) and 3-Iodo-L-tyrosine (0.1 and 1 mM). Nonlinear regression analysis to determine the pEC50 was carried out using GraphPad Prism (GraphPad Software, version 6.0, San Diego, CA, USA) with the constraint that *F*=0. All concentration–response data were evaluated for a fit to a logistics function in the form: E=E_max_/([1+ (10c/10x)n]+F, where E represents the increase in response contractile induced by the agonist, E_max_ is the effect agonist maximum, c is the logarithm of concentration of the agonist that produces 50% of E_max_, x is the logarithm of the concentration of the drug; the exponential term, n, is a curve fitting parameter that defines the slope of the concentration–response line, and F is the response observed in the absence of added drug. The values of EC50 data represent the mean±s.e.m. Values of E_max_ were represented by mN.

### Electrical-field stimulation-induced aorta contractions

The aortic rings were submitted to EFS at 60 V for 30 s, at 16 Hz in square-wave pulses, 0.3 ms pulse width and 0.1 ms delay, using a Grass S88 stimulator (Astro-Medical, Industrial Park, RI, USA). Electrical-field simulations were performed with and without L-NAME (100 µΜ), SCH-23390 (1 μM), risperidone (1 μM), quetiapine (1 μM), haloperidol (1 and 3 μM), salsolinol p(100 μM) and 3-Iodo-L-tyrosine (1 mM). Potassium chloride (KCl, 80 mM) was added at the beginning and at the end of the experimental protocols to ensure the tissue integrity after EFS.

### LC-MS-MS analysis

Two aortic rings per animal (15 mm) from *Chelonoidis carbonaria*, one endothelium-intact and another endothelium-denuded aortic ring were suspended in 5 ml organ baths containing Krebs-Henseleit's solution and O_2_/CO_2_ mixture at 27°C. The removal of endothelial cells was done mechanically by gently rubbing the arteries with forceps.

The basal release of dopamine, noradrenaline and adrenaline in Henseleit's solution was measured by LC-MS-MS following a 30 min incubation period. The dopamine, noradrenaline and adrenaline concentrations in the Krebs-Henseleit solution were determined by liquid chromatography coupled to tandem mass spectrometry (LC-MS/MS). The extraction procedure was similar to that described for extracting methyldopa from plasma (Oliveira et al., 2002). Briefly, 100 µl of the internal standards (dopamine-d3, noradrenaline-d6 and adrenaline-d6 at 100 ng/ml) were added to the Krebs’ solution (2 ml) followed by 1.5 ml of deionized water. After vortexing for 10 s, 100 mg of Al_2_O_3_ was added and left for incubation for 20 min in an orbital agitator (Centrifuge 5810/5810 R). The tubes were then centrifuged at 2000 g for 4 min at 4°C and the supernatant discarded. The residue was washed four times with 2 ml of deionized water. After the final washing, 200 µl of a solution containing trifluoroacetic acid 0.1% in HCN/H2O (60/40 l; v/v) were added. After vortexing for 40 s, the Eppendorf tubes were centrifuged for 2000 ***g*** for 5 min and the supernatant transferred to the vials for injection. The samples were analyzed by liquid chromatography coupled to a triple quadrupole mass spectrometer, LCMS-8050 (Shimadzu, Kyoto, Japan). The separation of catecholamines was performed on a 100×4.6 mm Lichrospher RP-8 column (GL Sciences Inc., Tokyo, Japan) using acetonitrile/water (5/95, v/v) with 0.1% formic acid as mobile phase at a flow rate of 0.4 ml/min. The mass spectrometer operated in positive electrospray ionization mode (ES+) for catecholamine detection. The analyses were executed in selected Multiple Reaction Monitoring (MRM) detection mode. This method has been fully validated, and the results reported elsewhere (Britto-Junior et al., 2020c).

### Data analysis

Data are expressed as mean±standard error of mean (s.e.m.) of the number of experiments. In the pharmacological experiments, the number of experiments in expressed as x/y, where x represents the number of aortas (animal) and y the number of rings employed in the experiment. The contractions were quantified in milli-Newtons (mN). A *P* value <0.05 was considered significant. When paired contractions were used, for example, in the absence and in the presence of an antagonist/inhibitor (the first contraction being the control response), Student's paired *t*-test was employed for statistical analysis. When one ring was used as the control response, and another ring was incubated with an antagonist/inhibitor, Student's unpaired *t*-test was used. For E_max_ analysis and pEC50, unpaired Student's *t*-test was used.

### Immunohistochemistry

Immunohistochemistry was performed manually. Briefly, 4-µm sections of formalin-fixed, paraffin-embedded samples of Chelonoidis aorta (*n*=4) were deparaffinized in xylene and rehydrated in a series of ethanol baths of increasing concentration. They were then incubated in citrate buffer at pH 6.0 in a steamer set for 40 min (at approximately 95°C). Following this, the sections were incubated for 2hs at room temperature (25°C) with a mouse monoclonal anti-chromogranin A antibody (clone DAK-A3, code M0869, 1:700, Dako/Agilent). Subsequently, these sections were incubated with the NovoLink Max Polymer Detection System (Novocastra/Leica Biosystems), following the manufacturer’s instructions, and using diaminobenzidine (liquid DAB, DakoCytomation, Carpenteria, CA, USA) as a chromogen (which renders a brown precipitate at the antibody binding site). Finally, the sections were counter-stained with Harris’ hematoxylin and cover-slipped in Entellan. Formalin-fixed, paraffin-embedded sections of a neuroendocrine tumor and a sample of normal intestinal (colonic) mucosae were used as positive controls for the presence of chromogranin A. All slides were examined using a trinocular Eclipse E200 microscope (Nikon, Tokyo, Japan) coupled to a 10MP CMOS digital camera (Amscope, USA).
